# Mass Generation, Neuron Labeling, and 3D Imaging of Minibrains

**DOI:** 10.3389/fbioe.2020.582650

**Published:** 2021-01-07

**Authors:** Subashika Govindan, Laura Batti, Samira F. Osterop, Luc Stoppini, Adrien Roux

**Affiliations:** ^1^Tissue Engineering Laboratory, Haute école du paysage, d’ingénierie et d’architecture de Genève, Haute école spécialisée de Suisse occidentale (HEPIA HES-SO), University of Applied Sciences and Arts Western Switzerland, Geneva, Switzerland; ^2^ARIMA Lifesciences PVT Ltd., Chennai, India; ^3^Wyss Center for Bio and Neuroengineering, Geneva, Switzerland; ^4^Swiss Center for Applied Human Toxicology (SCAHT), Bern, Switzerland

**Keywords:** 3D cell culture, 3D imaging, tissue clearing, neuron labeling, human iPSC derived models, brain spheroids, tissue engineering

## Abstract

Minibrain is a *3D brain in vitro* spheroid model, composed of a mixed population of neurons and glial cells, generated from human iPSC derived neural stem cells. Despite the advances in human 3D *in vitro* models such as aggregates, spheroids and organoids, there is a lack of labeling and imaging methodologies to characterize these models. In this study, we present a step-by-step methodology to generate human minibrain nurseries and novel strategies to subsequently label projection neurons, perform immunohistochemistry and 3D imaging of the minibrains at large multiplexable scales. To visualize projection neurons, we adapt viral transduction and to visualize the organization of cell types we implement immunohistochemistry. To facilitate 3D imaging of minibrains, we present here pipelines and accessories for one step mounting and clearing suitable for confocal microscopy. The pipelines are specifically designed in such a way that the assays can be multiplexed with ease for large-scale screenings using minibrains and other organoid models. Using the pipeline, we present (i) dendrite morphometric properties obtained from 3D neuron morphology reconstructions, (ii) diversity in neuron morphology, and (iii) quantified distribution of progenitors and POU3F2 positive neurons in human minibrains.

## Introduction

*In vitro* culture models have been integral in studying aspects of brain development and function in real time. The advent of human induced pluripotent stem cells (iPSCs) has accelerated the development of miniature *in vitro* 3D human brain models for the purpose of disease modeling, drug testing and molecular screening. 2D *in vitro* modeling of the human brain involves either (i) derivation of neural progenitor cells from primary human sources at gestational ages, (ii) differentiation of embryonic stem cells or iPSCs to neural progenitors or (iii) forced expression of neuronal transcription factor to transdifferentiate iPSCs to neurons (Pang et al., 2011; Zhang et al., 2013). These differentiated neurons or neural progenitor cells can then be used to generate 3D brain *in vitro* models (containing different types of CNS neurons and glial cells) by (i) culturing on low adhesion cell culture surfaces under agitation, (ii) hanging drop techniques, and (iii) seeding cells at high compaction ratio which are variedly termed as neurospheres, brain spheroids, brain micro physiological systems and brain aggregates models (Hogberg et al., 2013; Smirnova et al., 2016; Krencik et al., 2017; Pamies et al., 2017). Alternatively, embryoid bodies, i.e., spherical ensemble of human iPSCs or embryonic stem cells (ESCs) can be directed into neuronal differentiation that leads to formation of 3D cultures with the cytoarchitecture of the cerebral cortex (Pasca et al., 2015; Otani et al., 2016) which are termed either as spheroids or brain microphysiological system. The more advanced and complex 3D brain *in vitro* models are brain organoids that have been recently been developed by Kadoshima et al. (2013) and Lancaster et al. (2017) which recapitulated the self-organizing principle of forebrain structures while differentiating iPSC to the neuronal lineage using non-guided differentiation protocol.

Despite the advances in the field of 3D brain *in vitro* models, the smaller size of these models presents some challenges in performing classical histological and imaging techniques. On the other hand, some 3D *in vitro* brain models such as the cortical organoids are rather bigger in size but offer challenges for multiplexing for large scale screening studies and necrose relatively rapidly over a few months (Wang, 2018). Here, we present a protocol optimized for generating a brain spheroid model termed as minibrains, where the size of the spheroids and subsequent imaging techniques are optimized for large-scale screening studies at affordable cost, time and labor efficiency.

### Minibrains: Miniature 3D Brain Spheroids Generated From Neural Stem Cells Derived From Human iPSCs

**FIGURE 1:**
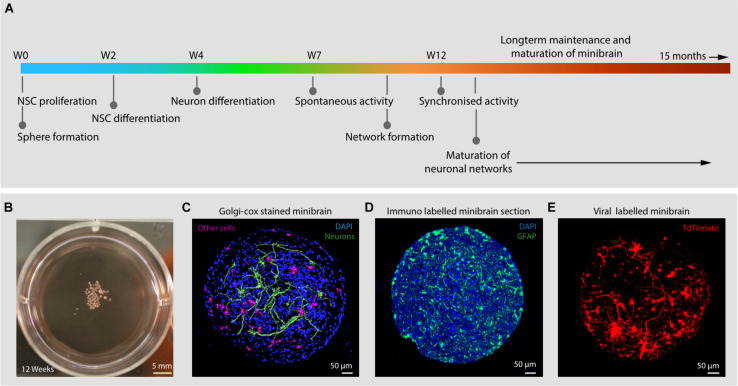
Introduction to minibrains. **(A)** Schematics show the timeline of processes involved in minibrain development. **(B)** Shows 12-week-old minibrains in a 6-well plate. **(C)** Volume rendered image of Golgi-Cox and DAPI stained (a nuclear stain) 12-week-old whole minibrain showing neurons with elaborate projections (color coded in green) and cells with short protrusions (color coded in pink) (see Supplementary Video 1). **(D)** Shows microtome cut 12-week-old minibrain section stained with GFAP, a marker of glial cells and DAPI. **(E)** Shows volume rendered image of viral labeled 12-week-old whole minibrain showing tdTomato (a fluorescent protein) labeled projection neurons. W, Week of minibrain generation.

**FIGURE 2:**
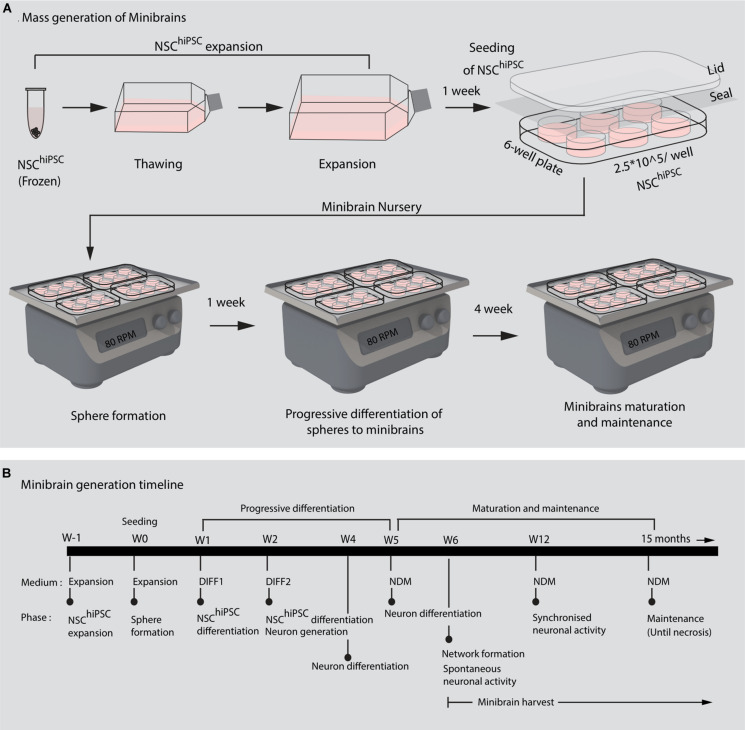
Mass generation of minibrains. **(A,B)** Shows the processes and timeline of establishing and maintaining minibrain “nursery” NSC^hiPSC^, Neural stem cells derived from human induced pluripotent stem cells; W, Week of minibrain generation; DIFF1, Differentiation 1 medium; DIFF2, Differentiation 2 medium; NDM, Neuron differentiation medium.

**FIGURE 3:**
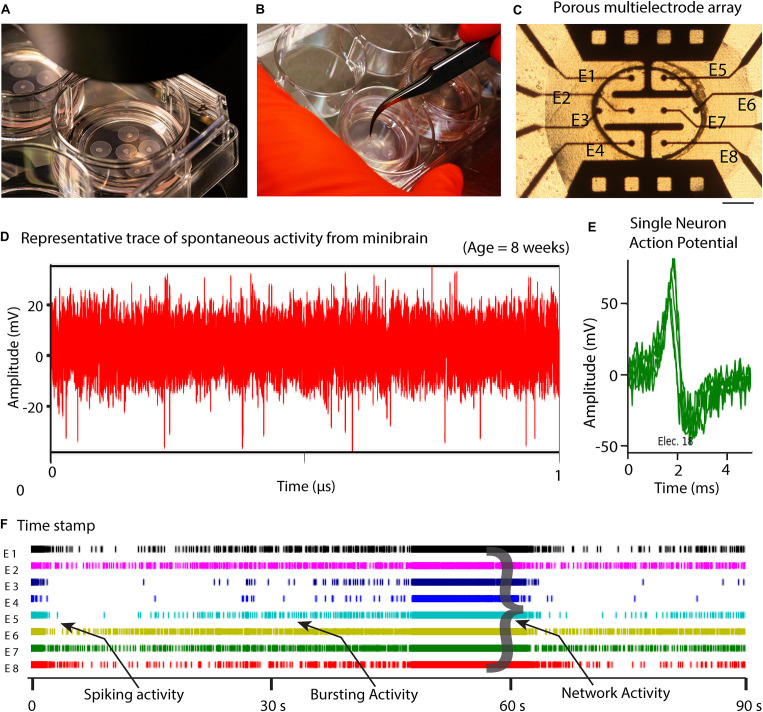
8-week-old minibrain displays network activity. **(A)** Shows minibrains on ALI (Air-liquid interface) culture **(B)** Shows ease of handling of minibrains on ALI culture **(C)** Minibrain on ALI culture integrated on MEA chip for performing neural activity measurement, Scale bar = 100 μm. **(D)** Typical time series of spontaneous activity recorded from one electrode from one 8-week-old minibrain. **(E)** Overlap of several action potentials of a single neuron recorded from a single electrode and identified using spike sorting analysis. **(F)** Timestamps indicating biological events was detected in real time by thresholding (± 6 standard deviation of the noise) where spike, bursting and network activities could be observed using spike sorting analysis. Data shown here corresponds to neuronal activity of one minibrain (*N* = 1). ALI, Air-liquid interface; E, Electrode; V, Volts; s, seconds.

**FIGURE 4:**
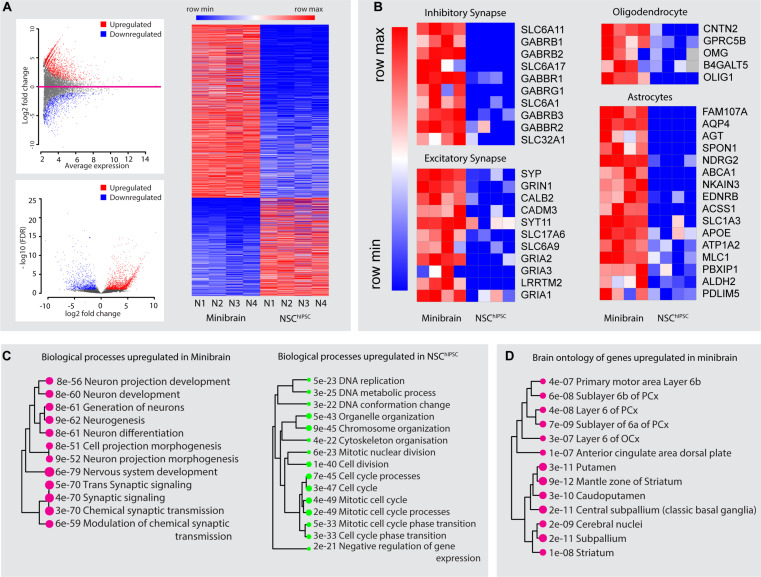
Gene expression profiling of minibrains. **(A)** Differentially regulated genes between minibrains and NSC^*hiPSC*^ were identified with a minimum fold change of 2 (top left), and false discovery rate (FDR) of 1 (bottom left). Heatmap of the expression of upregulated and downregulated genes in the minibrain compared to NSC^hiPSC^ (right) (*N* = 4). Multiple minibrains from 4 different batches were used. NSC^hiPSC^ from different batches were used as replicates. **(B)** Heatmap of expression profile of select genes representing inhibitory synapses, excitatory synapses, oligodendrocytes and astrocytes in minibrains and NSC^hiPSC^. Heatmap shows enrichment of these genes in minibrain, confirming the presence of inhibitory neurons, excitatory neurons, oligodendrocytes and astrocytes. **(C)** Shows biological process upregulated/enriched in minibrain (left) and downregulated in the minibrain/enriched in the NSC^hiPSC^ (right). The numerical value and size of the circle corresponds to the *p*-value of the biological process calculated by gene ontology enrichment analysis **(D)** Shows enrichment of distinct brain region gene expression profiles as per allen brain atlas database. The numerical value and size of the circle corresponds to the *p*-value of the brain region enrichment analysis. NSC^hiPSC^, Neural stem cells derived from human induced pluripotent stem cells.

In this study, we present minibrains, a brain spheroid model generated by non-directed differentiation of neural stem cells derived from human iPSCs (NSC^hiPS^) (Figures 1, 2) (protocol was modified and adapted from Sandström et al., 2017). By 8 weeks minibrains display synchronized neural networks (Figure 3; Sandström et al., 2017). Genes characteristic to neurons, oligodendrocytes and astrocytes were expressed in the minibrain. Transcriptomic analysis of minibrain vs NSC^hiPS^ revealed activation of biological process such as synaptic signaling, neuron morphology projection, neuron differentiation and neurotransmitter release. Minibrain expressed genes that is typically enriched in cortical regions such as striatum, sub pallium, layer 6 of motor cortex, piriform, anterior cingulate and occipital cortex (Figure 4).

**FIGURE 5:**
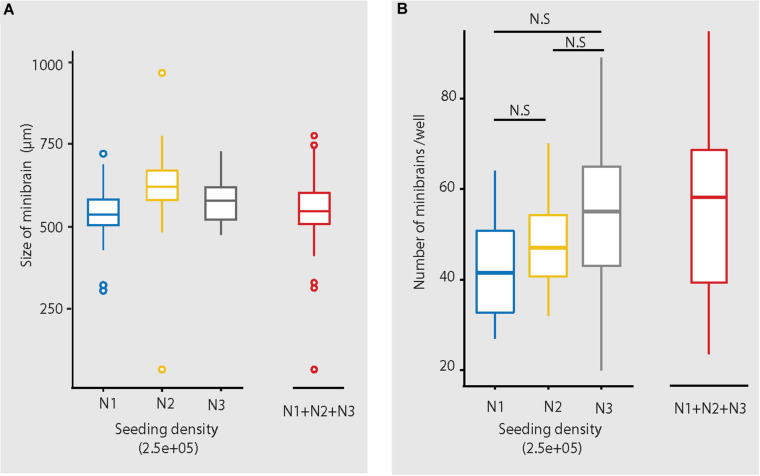
Size and number of minibrains generated using the protocol. **(A)** Shows the distribution of size of minibrains older than 8 weeks across three different batches (N1, N2, and N3) (with seeding density of 2.5e5 cells per well) as a whisker plot. Mean ± Standard deviation (SD) of N1, N2, and N3 are 546.80 ± 62.40, 623.61 ± 68.52 and 584.73 ± 79.02, respectively. Collective mean ± SD of all the three batches is 550.64 ± 75.19. N1 = 199 minibrains, N2 = 304 minibrains, N3 = 93 minibrains. **(B)** Shows the number of minibrains (more than 8 weeks old) generated per well in a 6-well plate across three different batches (N1, N2 and N3 with seeding density of 2.5E5 cells per well) as a whisker plot. The number of minibrains generated across three batches are not significantly different (*p* = 0.02 8) as per Kruskal Wallis statistical test. Mean ± SD of N1, N2, and N3 are 42.93 ± 12.00, 47.75 ± 8.37, 52.47 ± 16.38, respectively. Collective mean ± SD all the three batches is 48.97 ± 12.68. N1 = 15 wells, N2 = 45 wells and N3 = 60 wells.

The size, simplicity and cost efficiency of generating minibrains make them an ideal choice for mass production for the purpose of large-scale screening and modeling studies (Figure 2 and Supplementary Table 1). The average size of minibrains is around 550.64 (±) 75.19 μm and around 50–100 minibrains can be generated and maintained in one well of a 6-well plate (Figure 5 and Table 1). The cost of generating approximately 100 minibrains is 33.50 CHF, the cost of generating and maintaining a “nursery” of approximately 6,000 minibrains for about 1-year costs about 3740 CHF. The smaller size of minibrains, allows efficient diffusion of nutrients to cells until the center of the minibrains, reducing the occurrence of necrosis and maintaining minibrain for up to 15 months and above. Minibrains can subsequently be maintained on an air-liquid interface (ALI) to facilitate brain on chip methodologies for neuronal network activity measurement using micro-electrode-array integrated biochip (Figure 3).

**TABLE 1:** Plating guideline for Minibrain formation.

	No. of cells	Medium volume	Average number of spheres	Requiered volume of cell suspension
6-well	1*10^6^	3 ml	95 ± 34 (*N* = 12 wells)	2*10^6^/X
6-well	2.5*10^5^	3 ml	49 ± 13 (*N* = 129 wells)	5*10^5^/X
24 well	2*10^4^	500 μl	1 or 2	4*10^5^/X

*X, number of living cells/ml in cell suspension.*

Labeling neurons and subsequent morphological reconstruction of the neurons provides information about the dendrite morphometric and axonal properties that are crucial for network establishment. These properties can give valuable insight in large scale disease modeling and drug testing screens (DePoy et al., 2014; Miguéns et al., 2015; Cheng et al., 2017; Martínez-Cerdeño, 2017; Patnaik et al., 2020). Much is yet to be understood as to how human neuron morphology is driven in *3D brain in vitro* models where all connectivity pathways are miniaturized. Labeling of live neuron morphology involves artificial gene transfer techniques that enable expression of fluorescent reporters under the regulation of selective promoters. In previous studies, neurons were labeled in live brain organoids either through electroporation or viral infection of organoid slices, which are laborious processes (Lancaster et al., 2017; Giandomenico et al., 2019). In our pipeline, we implement a novel strategy to label projection neurons with tdTomato fluorescence reporter in the minibrains using retrograde adeno associated viral particles (AAVrg) (Figures 7A–C). Sparse neuronal labeling, together with a high signal to noise ratio of the labeled neurons, enabled 3D reconstructions of distinct neurons within the minibrain (Figures 7B,C). For the first time, we show reconstruction of single neuron morphologies from an 3D *in vitro* brain model, which would allow us in the future to understand neuron differentiation, diversity and connectivity in 3D brain *in vitro* models (Figures 7C–F and Supplementary Figure 3).

**FIGURE 6:**
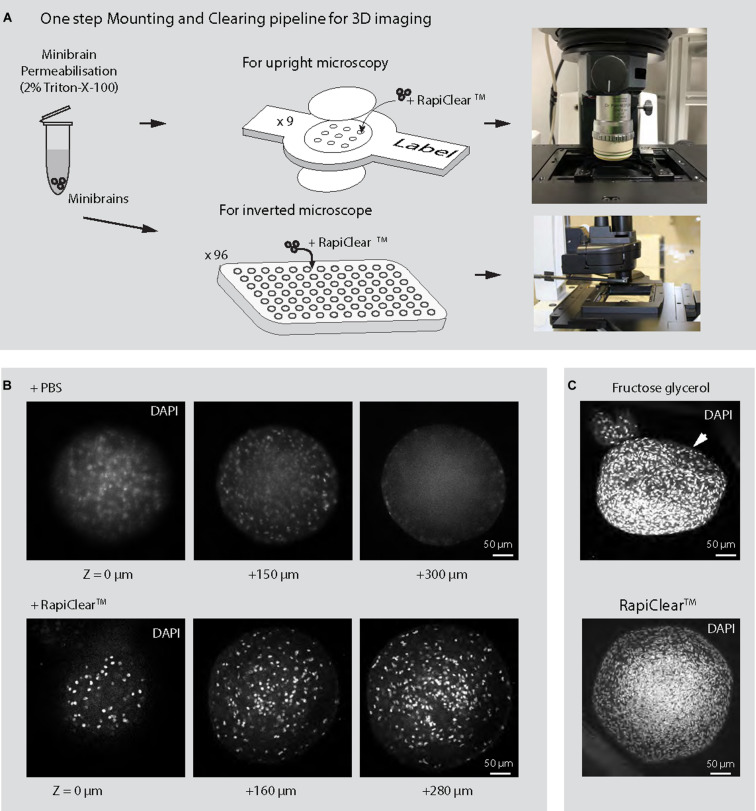
3D imaging of minibrains. **(A)** Shows the pipeline for imaging minibrains using upright and inverted confocal microscopes. **(B)** Shows the representative 2D optical sections of 10-week-old minibrains stained with DAPI (a nuclear stain) (*N* = 3 minibrains) without clearing (top) and upon clearing with RapiClear^TM^ at different Z imaging depth. **(C)** Shows morphological changes in 10-week-old minibrains upon clearing with fructose glycerol (top) and intact morphology while using RapiClear^TM^ (bottom). The images are representative maximum intensity projection of approximately 600 μm confocal Z-stack of the minibrain (*N* = 5 minibrains).

**FIGURE 7:**
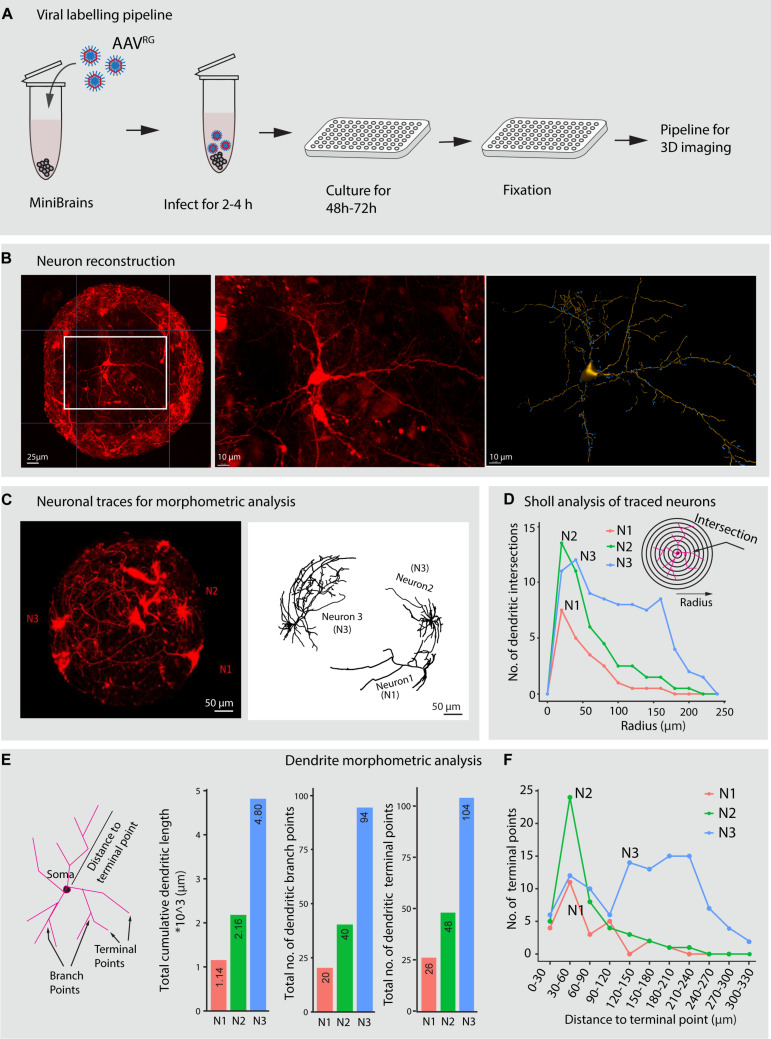
Viral labeling and neuron morphology reconstruction in minibrains. **(A)** Shows a multiplexable viral infection pipeline to label and 3D reconstruct projection neurons in minibrains. **(B)** Shows representative maximum intensity projection of approximately 300 μm confocal Z-stack of minibrain labeled with tdTomato by AAV^RG^ infection (left), an inset zoom on one neuron (center) and the 3D segmentation of the neuron using filament pipeline in Imaris (right) (Age of minibrain = 7 months, *N* = 20 minibrains). **(C)** Shows a representative maximum intensity projection of approximately 600 μm confocal Z-stack of a whole minibrains with tdTomato labeled neurons (Age of minibrain = 3 months) (left), neuron morphology traces of 3 selected neurons (N1, N2, and N3) from the same minibrain segmented by Imaris software (right). **(D)** Sholl analysis of dendrites of the three selected neurons N1, N2, and N3 within the one minibrain in **(C)**. **(E)** Shows dendrite morphometric features (left) and quantification of three properties across three neurons. The bar graphs from left to right show cumulative summation of all dendritic branch length, dendritic branch points and dendritic terminal in three traced neurons shown in **(C)**. **(F)** Shows distribution of the number of the dendritic terminal points at different distances from the soma across the 3 neurons N1, N2, and N3 traced in **(C)**. AAV^RG^, Adeno associated virus retrograde serotype; h, hours post infection. Neuron morphological analysis performed from three more minibrains are presented in Supplementary Figure 3.

**FIGURE 8:**
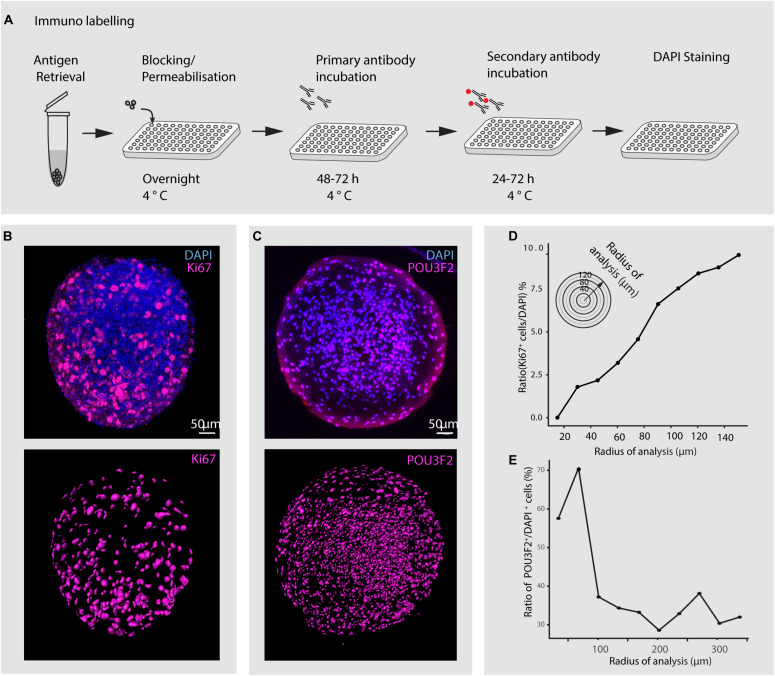
Immunohistochemical analysis of minibrains. **(A)** Shows a multiplexable immunohistochemistry pipeline for minibrains. **(B)** Shows a representative rendered maximum intensity projection of 3D whole minibrain image stained for Ki67 (progenitor marker) and DAPI (top), subsequent segmentation of Ki67 cells (bottom) by Imaris pipeline. Age = 1 week, *N* = 9 minibrains. **(C)** Shows a representative maximum intensity projection (15 μm) of whole minibrain image (age of minibrain = 16 weeks) stained for POU3F2 (excitatory neuronal marker) and DAPI (top), subsequent segmentation of POU3F2+ neurons cells (bottom) by Imaris pipeline. The images are representative of multiple tests *N* = 9 minibrains. Age = 12 weeks. **(D)** Graph shows the distribution of the ratio of Ki67 expressing progenitors to DAPI stained nuclei across minibrain (*N* = 1 minibrain), illustrating the increase in progenitor density toward the periphery in the minibrain in **(B)**. **(E)** Graph shows the distribution of the ratio of POU3F2^+^ expressing neurons to DAPI stained nuclei across one minibrain, illustrating neurons enriched in the center of the minibrain in **(C)** (*N* = 1 minibrain). Analysis in **(D,E)** was performed only in one minibrain to illustrate an example of cell distribution analysis using 3D imaging. h, hours post staining.

3D imaging techniques are critical for reconstructing whole neuron morphology, assessing anatomical distribution of cell types and their interaction across *3D brain in vitro* models. Here we present a novel 3D imaging pipeline that allows imaging of whole *3D brain in vitro* models. 2D imaging methodologies require slicing of *3D brain in vitro* models which can be laborious, time consuming and leads to loss of tissue given the smaller sample size. In contrast, our 3D imaging pipeline relies on easily multiplexable one step non-invasive tissue clarification technique on whole minibrains. We tested multiple tissue clarification protocols on our minibrains. While active CLARITY techniques are too harsh on the minibrains, passive CLARITY technique required embedding the minibrains in agarose gel, a cumbersome process when processing multiple minibrains. Tissue clarification method using fructose-glycerol solution lead to distortion of the minibrain morphology (Figure 5C). On the other hand, RapiClear^TM^, a commercial tissue clearing agent, allowed direct mounting of minibrains without loss of morphology and best signal preservation for viral labeling, immunohistochemistry and Golgi-Cox staining (Figures 6–8 and Supplementary Videos 1–4). We achieved efficient clarification by permeabilizing fixed minibrains using Triton X-100 before clearing with RapiClear^TM^ (Figures 6A,B). RapiClear^TM^ allows preservation of mounted minibrains at 4°C and −20°C for long term storage. The cleared minibrains were compatible for both confocal and light sheet microscopy (Supplementary Videos 1–4 and Figure 6). For high resolution imaging using upright microscopy, we have used an upright confocal imaging and designed sample holders and microscopy support to facilitate whole mount minibrain imaging in high refractive index (RI) solution, while multiplexing up to 9 samples (Supplementary Figure 2). The long travel distance of the immersion objectives (5.6 mm) allowed us to scan through the whole thickness of the cleared samples (Figure 6 and Supplementary Videos 1, 3). The minibrains can be imaged until a depth of 150–250 μm using inverted microscopy allowing multiplexing up to 96 or 384 samples by using multi-well imaging plates (Supplementary Video 5).

We present here a step-by-step methodology for generation and maintenance of minibrain nurseries, ALI maintenance of minibrain, projection neuron labeling, optimized whole minibrain immunohistochemistry, one step mounting clarification, 3D imaging and design of imaging accessories that facilitate 3D imaging using confocal microscopy on cleared minibrains. We present dendrite morphometric properties, diverse reconstructed neuron morphologies, distribution of progenitors and POU3F2^+^ neurons in our minibrain (Figures 7, 8). Our novel pipeline is designed to facilitate the usage of minibrains as an *in vitro* 3D human neuronal model for large scale modeling and mass screening studies.

## Materials

### Reagents

GelTrex^TM^ (ThermoFisher, #A1413301)

Stempro^TM^ NSC SFM kit (ThermoFisher, #A1050901) containing

KnockOut^TM^ DMEM/F-12 medium

StemPro^TM^ Neural Supplement

FGF-basic (AA 10–155) Recombinant Human Protein

EGF Recombinant Human Protein

GlutaMAX^TM^ Supplement (ThermoFisher, #35050038)

B27^TM^ Plus Neuronal Culture System (ThermoFisher, #A3653401) containing

Neurobasal^TM^ Plus

B-27 Plus Supplement (50X)

Accutase (ThermoFisher, #00-4555-56)

StemPro^TM^ hESC SFM (ThermoFisher, #A1000701) containing

DMEM/F12 + GlutaMAX^TM^

Bovine serum albumin (BSA) 25%

Stempro^®^ hESC Supplement

Brain-Derived Neurotrophic Factor (BDNF) Recombinant Human Protein (ThermoFisher, #PHC7074)

Glial-Derived Neurotrophic Factor (GDNF) Recombinant Human Protein (ThermoFisher, #PHC7044)

Dibutyryl cyclic AMP (AMPc) (Merck Sigma, #D0627)

2-phospho-Ascorbic Acid (Merck Sigma, #49752)

RapiClear^TM^ 1.47 (SUNJin Lab, #RC147001)

DAPI (4′,6-Diamidino-2-Phenylindole, Dihydrochloride) (Invitrogen, #D1306)

Triton X-100 (Merck Sigma Aldrich, #T8787-50ML)

Tween 20 (Merck Sigma Aldrich, #P9416-50ML)

1X Dulbecco’s PBS (DPBS) (ThermoFisher, #14040133)

Pierce^TM^ 16% Formaldehyde (w/v), Methanol-free (ThermoFisher, #28906)

Trypan Blue Stain (0.4%) (ThermoFisher, #15250-061).

Purified Mouse Anti-Human Ki67 antibody (BD Bioscience, #556003)

Mouse Anti-Human POU3F2 antibody (DSHB, #PCRP-POU3F2-1A3-s)

Histodenz^TM^ (Sigma, #D2158)

Goat anti-mouse TRITC (Abcam, #ab6786).

### Plastics and Tools

6-well culture plate (Greiner Bio-One, #657 160)

96-well culture plate for imaging (Greiner bio-one, #655090)

384 well culture plate for imaging (Greiner bio-one, #781091)

24 well culture plate non-treated (Nunc, #144530)

Breathable plate sealer (Greiner Bio-One, #676051)

25 cm^2^ cell culture flask (T25) (Corning Falcon, #353109)

75 cm^2^ cell culture flask (T75) Flask (Corning Falcon, #353136)

175 cm^2^ cell culture flask (T175) Flask (Corning Falcon, #353112)

Sterile Hydrophilic PTFE membrane for tissue cultures, 2 mm diameter (named as « confetti ») (PTFE-005, HEPIA Biosciences)

Cell culture inserts for 6-well plate (Merck Millipore, #044003)

0.2 ml PCR tube (Thermoscientific, #AB-0784)

1.5 ml microfuge tubes (Eppendorf, #0030125150)

15 ml tube with conical bottom (Corning Falcon, #352096)

50 ml tube with conical bottom (Corning Falcon, #352070)

Pointe 10–20 μl cleanpack clearline sterile (Milian, #010320)

Pointe 20 μl cleanpack clearline sterile (Milian, #713178)

Pointe 200 μl cleanpack clearline sterile (Milian, #713179)

Pointe 1,000 μl cleanpack clearline sterile (Milian, #713180)

10 μl pipette (Rainin, #17014388)

20 μl pipette (Rainin, #17014392)

200 μl pipette (Rainin, #17014391)

1,000 μl pipette (Rainin, #17014382)

2 ml sterile aspirating pipets (Corning Falcon, #357558)

12 mm glass coverslips (SPL Life Sciences, #20012)

Luna Cell Counting Slides (Logos biosystems, #L12001)

Sample holder (HEPIA, Supplementary Figure 2).

### Cells

NSC^hiPS^ (Human Neural Stem Cells derived from the human induced pluripotent stem (iPS) cell line) (ThermoFisher, #A3890101).

### Virus

AAVrg-CAG-tdTomato (codon diversified), titer value ≥ 7 × 10^12^ vg/ml (Addgene, #59462-AAVrg).

Important: Virus must be handled in biosafety level 2 (BSL2) facility

Note: Upon receipt of the virus, prepare 5 μl aliquots on ice and store at −80°C.

### Equipment

CO_2_ resistant orbital Shakers (ThermoFisher, #88881102)

CO_2_ incubator (ThermoFisher, #371)

Confocal microscope (Zeiss, LSM880)

20X/1.0 water immersion objective, with adjustable RI correction collar (RI 1.42–1.48) (Zeiss, #421459-9972-000)

Confocal Microscope (Leica, TCS SPE-II) HC PL APO 10.0 × 0.30NA objective (Leica, #507902)

HCX PL FLUOTAR L 20X/0.40NA objective (Leica, #506242)

Specimen holder for cleared tissue imaging (HEPIA, Supplementary Figure 2)

Laminar hood (SKAN AG, #MSF120)

Centrifuge (Eppendorf, #5804 R)

Media Warmer (Lab Armor^TM^ Beads, #M706)

Elliptical shaker 3D Polymax 1040 complete (Heidolph Instruments, #543-42210-00)

Thermal cycler (MJ Research, #PTC-200)

Luna Automated Cell Counter (Logos biosystems, #L10001)

Inverted microscope (Zeiss, #Axiovert 25)

Laboratory vacuum pump (Milian, #886083)

Regine Horlogery watchmaker tweezers, type 7 (Beco Technic, #220337)

Mr. Frosty^TM^ Freezing Container (ThermoFisher, #5100-0001).

## Reagent Setup

### GelTrex (1:200)

Thaw 1 ml vial of GelTrex at 4°C overnight, without agitation (!!! Attention: Agitation will cause clumping).

Aseptically add 1 ml of GelTrex to 199 ml of cold KnockOut^TM^ D-MEM/F12 medium.

Can be stored at 4°C for up to 1 month.

### Stock Solutions of Supplements

Prepare stock solution for EGF, FGF, BDNF (20 ng/ml), GDNF (20 ng/ml), and 2phospho-Ascorbic Acid (20 mM) by resuspending the powder in DPBS, 0.1% BSA, prepare 20 μl or 100 μl aliquots, and freeze at −20°C. Aliquots can be kept for 2 years.

Prepare stock solution for Dibutyryl cyclic AMP (100 mM) by resuspending the powder in sterile water, prepare 20 μl or 100 μl aliquots and freeze at −20°C- Aliquots can be kept for 2 years.

### Expansion Medium

Prepare expansion medium by aseptically mixing components of Stempro^TM^ NSC SFM kit:

500 ml, KnockOut^TM^ D-MEM/F12

5 ml, GlutaMAX^TM^ Supplement

10 ml, StemPro^TM^ Neural supplement

This medium can be stored at 4°C for up to 3 months.

Before use, aseptically add 100 μl of EGF stock and 100 μl of FGF stock to 50 ml of the above media mix. This solution can be stored at 4°C for up to 1 week.

### Differentiation 1 Medium (DIFF1)

For 50 ml of medium aseptically mix

44.45 ml, D-MEM/F12 + GlutaMAX

3.6 ml, 25% BSA

1 ml, Stempro hESC Supplement

100 μl, BDNF stock solution

100 μl, GDNF stock solution

250 μl, Dibutyryl cyclic AMP stock solution

500 μl, 2phospho-Ascorbic Acid stock solution

Note: Do not overheat this medium (for a long time) because several factors (BDNF-GDNF) are sensitive to heat!

This medium can be stored at 4°C for 1 week, but it is best to prepare it fresh before use.

### Neuron Differentiation/Maintenance Medium (NDM)

Aseptically mix:

500 ml, Neurobasal^TM^ Plus 1 × 10 ml, 50x B27 Plus Supplement

1.25 ml, GlutaMAX^TM^ Supplement

This medium can be stored at 4°C for up to 3 months.

### Differentiation 2 Medium (DIFF 2)

Aseptically mix DIFF1 medium and NDM medium at 1:1 ratio.

Note: Do not overheat this medium (for a long time) because several factors (BDNF-GDNF) are sensitive to heat!

This medium can be stored at 4°C for 1 week, the best is to prepare it fresh before use.

### 4% PFA

Aseptically dilute 0.5 ml of 16% PFA to 1.5 ml of 1X DPBS.

Store at 4°C for up to 1 month.

### Post-fixation Rinse Buffer: DPBS-Tween

Dissolve 1 ml of Tween-20 in 1000 ml of 1X DPBS.

Store at 4°C for up to 6 months.

### Antigen Retrieval Buffer

Dissolve 1.5 g of sodium citrate (10 mM) in 450 ml water, adjust pH to 6 with 1 N HCl. Make up the final volume to 500 ml and add 450 μl of Tween 20.

Store at 4°C for up to 6 months.

### Blocking Buffer

Dissolve 1 g of BSA and 2 ml of Triton X-100 in 100 ml of 1X DPBS.

Store at 4°C for up to 6 months.

### Wash Buffer

Dissolve 2 ml of Triton X-100 in 100 ml of 1X DPBS.

Store at 4°C for up to 6 months.

### Primary Antibody Dilution

Dilute primary antibody in blocking buffer as per antibody manufacturer’s instructions. Always prepare the dilution freshly before use.

Dilute Ki67 antibody (BD Bioscience, #556003) at 1:25 dilution in blocking buffer.

Dilute POU3F2 antibody (DSHB, #PCRP-POU3F2-1A3-s) at 1:5 dilution in blocking buffer.

### Secondary Antibody Dilution

Dilute anti-mouse TRITC secondary antibody (Abcam, #ab6786) at 1:500 dilution in blocking buffer.

### DAPI Stock Solution

Make a 5 mg/ml DAPI stock solution by dissolving 10 mg in 2 ml deionized water.

Make sure to dissolve the powder completely by vortexing the tube.

Prepare aliquots and freeze at −20°C for long term storage. Once an aliquot is open, it can be kept at 4°C for 6 months.

## Methods and Protocols

### Generation of Minibrains

#### Thawing Frozen Aliquots of NSC^hiPS^

Before starting the experiment

1. Coat a T25 flask by adding 3 ml of GelTrex stock. Make sure it covers the entire surface area and incubate for 37°C for 1 h.
  Note: optionally the coated flasks can be stored at 4°C for 1 month and pre warmed to room temperature before use.
2. Warm 9 ml expansion medium in a 50 ml conical tube to room temperature and 5 ml expansion medium in a 15 ml conical tubes to 37°C.
3. Warm water bath to 37°C.
4. Thaw a vial containing 1 ml of NSC^hiPS^ quickly in a 37°C water bath for not more than 30 s.
5. Transfer 1 ml of the thawed NSC^hiPS^ to a 50 ml tube under the hood using a 1 ml micropipette.
6. Rinse the vial with 1 ml prewarmed expansion medium (at room temperature) and add the contents using a 1 ml micropipette to the cells in the 50 ml tube.
7. Slowly add 8 ml of prewarmed expansion medium (at room temperature) to the thawed cells in the 50 ml tube while mixing gently.
8. Centrifuge the thawed cells at 250 × g for 5 min at room temperature. A cell pellet will be visible after centrifugation.
9. Aspirate medium without disturbing the pellet using an 1 ml micropipette and resuspend the cell pellet in 5 ml expansion medium in the 15 ml conical tube at 37°C using an aspirating pipette.
10. Add 5 ml of the resuspended cells to a GelTrex coated T25 flask and spread evenly.
11. Every second day change the expansion medium.
12. When the cells reach 80% confluency (typically in 2–3 days), passage with Accutase as described in step 13.

#### Passaging and Expansion of NSC^hiPS^

Before starting the experiment: warm Accutase and expansion medium (aliquots or the whole bottle) at 37°C. Coat flasks with GelTrex as described in step 1.

13. Aspirate all medium from the flask and add 2 ml of Accutase. Incubate at room temperature for 1–2 min.
  Tip: Swirl the plate gently to better visualize the detachment of the cells.
14. To collect the cells, add 2 ml of expansion medium to the detached cells and collect in a 15 ml tube using an aspirating pipette.
15. Rinse the remaining cells with an additional 2 ml of expansion medium and collect in the same tube.
16. Centrifuge cells at 250 × g for 5 min at room temperature.
17. Aspirate medium and resuspend pellet in 2–5 ml of expansion medium (depending on the size of the pellet) using an aspiration pipette.
18. Count live cells per ml on cell counter
  a. Mix 10 μl of Trypan Blue and 10 μl cell suspension in an 0.5 ml microcentrifuge tube.
  b. Transfer 10 μl of the mix on a counting slide
  c. Insert the counting slide in the Luna Automated Cell Counter, adjust the focus and press “count.”
  d. Number of live cells/ml = Number of counted cells^∗^ 2
19. Calculate the volume of cell suspension needed for each T75 flask using the formula below.
  Number of cells required = 25^∗^10^3^ cells/cm^2^
  Surface area of T75 flask = 75 cm^2^
  Number of live cells/ml = X
  Volume of cell suspension required = Number of cells required ^∗^ surface area of culture plate/(X)
20. Add the calculated volume of cell suspension using aspiration pipette and 10 ml of prewarmed expansion medium to a new GelTrex coated T75 flask.
21. Culture the cells in an incubator at 37°C and 5% CO_2_ until they reach 80% confluency, typically in 2–3 days.

#### Week 0: Formation of Spheres (3D)

22. Prepare NSC^hiPS^ cell suspension and calculate the number of live cells per ml (X) as described in Step 13–18.
23. Calculate volume of cell suspension to be added based on the guidelines in Table 1 and as described in step 19.
24. Add calculated volume of cell suspension and required volume of medium using an aspiration pipette required based on the guidelines in Table 1.
25. Seal the plate with breathable adhesive paper by detaching the plastic layer protecting the paper and carefully covering the plate using the adhesive side. Make sure all wells are covered by the paper. Close the lid of the plate (Supplementary Figure 1A).
26. To form spheres, place the cells on an orbital shaker at 80 rpm and culture for 7 days at 37°C and 5% CO_2_.
  Note: Spherical nascent minibrains are apparent after 24 h and increase in size is visible after 7 days due to proliferation.
  Important!!! From this point on, the culture plates should always be sealed with adhesive paper while culturing minibrains in suspension.

#### Week 1: Differentiation Phase I

27. Position the plates at a slanted angle allowing minibrains to settle down, aspirate the medium gently from the top without losing any minibrains.
28. Add 2.5 ml of DIFF1 medium per well (for a 6-well plate). Culture the minibrains for 7 days at 80 rpm at 37°C and 5% CO_2_.

#### Weeks 2–4: Differentiation Phase II

29. Aspirate medium as described in step 27, add 2.5 ml of DIFF2 medium (for a 6-well plate). Culture the minibrains for 3 weeks at 80 rpm in the cell culture incubator at 37°C and 5% CO_2_. Change DIFF2 medium twice per week.

#### Week 5 Until 1 Year and Above: Shifting to Maintenance Phase of the Minibrains

30. Aspirate medium as described in step 27. Add 2.5 ml of NDM medium (for a 6-well plate). Change the NDM once per week and check the luminosity of the minibrain.

Troubleshooting**:** Minibrains can go through fusion upon shifting to maintenance medium (Supplementary Figure 1B). Shift one well of minibrains to the maintenance medium, check for fusion of minibrains after 24–48 h. If minibrains have fused in the test well, prolong differentiation phase II until the next medium change (1 week), repeat testing for fusion until minibrains stop to fuse before shifting to maintenance medium.

Troubleshooting: Health of the minibrains can be monitored by screening of necrosis in the minibrains (Supplementary Figure 1C). If a minibrain displays complete necrosis, discard the entire well of minibrains.

Note: Minibrains can survive for 15 months and more, with neuronal activity (data not included). The minibrains are termed as early minibrains starting from the 6th week, as that is when they display synchronized neuronal network activity. Mature activity is observed at 8 weeks.

### ALI Culture of Minibrain on Confetti

1. Add 1 ml of NDM medium to a 6-well plate.
2. Place cell culture insert on the 6-well plate and place the confetti on the insert, enabling absorption of medium.
3. Warm the medium in the plate in the cell culture incubator for 30 min.
4. Set a 1 ml pipette at 20 μl and transfer one sphere onto the center of a confetti (check the sphere for luminosity before transfer).
5. Add 5 ml of water in the spaces between the well, to ensure humidity as the plates will not be sealed with breathable adhesive paper.
6. Change the medium twice per week by removing the maximum by aspiration and add 1 ml of NDM medium.

Note: Monitor necrosis under a light microscope to monitor health of minibrains.

### Viral Labeling of Projection Neurons in Live Minibrains

Viral labeling must be done in biosafety level 2 equipped facility under a hood.

Before starting the experiment:

1. Prepare an ice box and place the viral aliquots in the ice to thaw.
2. Set the centrifuge temperature to 4°C.
3. Warm 5 ml of media in a 15 ml conical tube in the cell culture incubator.
4. Centrifuge the virus at 100 × g for 10 minutes at 4°C, to avoid loss of virus and any spill.
5. Cut a 200 μl tip and transfer 3–4 minibrains to a 1.5 ml microcentrifuge tube.
6. Remove the excess media.
7. Add exactly 30 μl of media in each tube, let the minibrains settle to the bottom.
8. Add 0.5 μl of virus (3.5 × 10^9 particles) in each tube.
  Note: The pipette tip must contact the minibrain while adding the virus. Do not mix the tube after the addition of the virus.
  Using higher concentration of virus leads to more neurons labeled making it difficult to reconstruct single neuron morphologies.
9. Incubate the minibrains with virus in the cell culture incubator at 37°C and 5% CO_2_ for 2–4 h.
10. In the meantime, add 200 μl NDM medium to a 96-well plate and incubate at 37°C and 5% CO_2_.
11. Cut the tip of a 200 μl pipette tip and transfer 1 minibrain per well in the prepared 96-well plate and culture the minibrains for a minimum of 48 h.

### Immuno-Histochemical Staining of Minibrains

#### Minibrain Fixation

1. Remove media from the wells and add 100 μl of 4% PFA for a 96-well plate, 1 ml of PFA for a 24-well plate and 3 ml of PFA for a 6-well plate and incubate for 45 min at room temperature.
2. Wash the minibrains at room temperature by rinsing with DPBS-Tween for 10 min at 60 rpm on the elliptical shaker. Repeat two more times.

#### Antigen Retrieval

3. Transfer minibrains to a PCR tube using a cut 200 μl tip. Remove excess DPBS-Tween, add 100 μl of antigen retrieval buffer and incubate at 95°C for 1 h in a PCR cycler.
4. Wash the minibrains at room temperature by rinsing with wash buffer for 5 min at 60 rpm on the elliptical shaker. Repeat one more time.

#### Permeabilization and Blocking

5. Using a cut 200 μl tip transfer minibrains to a 96-well plate and remove excess buffer.
6. Resuspend the minibrains in 70 μl of blocking buffer and incubate for minimum 4 h at room temperature or overnight at 4°C on a rocker at 80 rpm.

#### Primary and Secondary Antibody Labeling

7. Remove the blocking buffer and add 70 μl of primary antibody dilution. Incubate at 4°C on a rocker at 80 rpm for 48–72 h.
8. Remove the primary antibody, add 70 μl of wash buffer, incubate for 30 min on a rocker at 80 rpm. Repeat two more times.
9. Remove the wash buffer and add 70 μl of secondary antibody dilution. Incubate at 4°C on a rocker at 80 rpm for 24–48 h, protected from light.
  Note: From this point on, protect the samples from light to avoid bleaching of the fluorescence.
10. Remove the secondary antibody and add 70 μl of wash buffer and place in a rocker at 80 rpm for 10 min.

#### Nuclei Staining With DAPI

11. Dilute DAPI stock solution 1:1,000 in wash buffer and incubate the minibrains for a minimum of 1 h.
12. Remove the DAPI solution, add 70 μl of wash buffer and place on a rocker at 80 rpm for 30 min. Repeat 2 more times.

### Minibrain Preparation for Microscopy

#### Permeabilization of Minibrain for Tissue Clearing

1. If the minibrains are fixed but were not processed further for immunohistochemistry, incubate minibrains with wash buffer for 2 h.

#### Mounting and Clearing Minibrains for 3D Confocal Imaging Using Upright Confocal Microscopy

2. In the meantime, prepare the inserts for confocal imaging as shown in Figure 6 and Supplementary Figure 2. Peel the adhesive protector from the middle on one side of the imaging insert and seal the open well holes on one side using an 18 mm glass cover slip.
3. Cut the tip of a 200 μl pipette tip, transfer minibrains in the wells of the imaging insert, remove excess buffer. One well can hold as much as 4–5 minibrains.
4. Add 20–30 μl of RapiClear^TM^ to the well as shown in Supplementary Figure 2. Make sure wells are completely filled.
5. Remove excess RapiClear^TM^ outside the wells using a 20 μl pipette.
6. Peel the adhesive tape in the middle and seal the wells of the imaging insert using another 18 mm coverslips.
7. Incubate the minibrains mounted with RapiClear^TM^ for 24 h at room temperature, protected from light.
  Note: Optionally lift the minibrains using a pipette tip before sealing the top of the imaging insert. This allows easier location of the minibrains during confocal imaging.

#### Minibrain Preparation for 3D Imaging Using Inverted Fluorescence Microscopy

8. Transfer permeabilized minibrains to an imaging grade 96 or 384-well plate.
9. Remove any remaining buffer completely and add 80 μl of RapiClear^TM^ per well for a 96-well plate and 50 μl of RapiClear^TM^ per well for 384-well plate. Incubate in RapiClear^TM^ for 18–24 h and proceed for imaging.

### Imaging Minibrains

#### Imaging Using Upright Confocal Microscopy

1. Mount the 20X/1 water immersion objective, with adjustable RI correction collar (RI 1.42–1.48) onto the LSM-880 confocal microscope (Zeiss).
2. Prepare the mounting insert by cleaning it. Place the imaging insert as in Supplementary Figure 2 and secure the insert tightly using the screws on the image insert holder.
3. Fill the mounting insert with Histodenz solution (RI = 1.46).
  Note: if the imaging insert is not secured using the brace, the insert will start lifting up while imaging.
4. Perform multi-tile and Z-stack imaging, using confocal or Airy scan module, with step-size of 3 μm for neuron reconstructions, and 10 μm for profiling the minibrain for signal.
  Note: Alternatively, lightsheet microscopy technique can be used to image cluster of minibrains all at once, by using a lightsheet microscope optimized for large cleared samples (Clarity Optimized Light-sheet Microscope – COLM) (Supplementary Video 2).
  Note: To perform image analysis and 3D rendering on the z-stack use FIJI or Imaris software (filament tracer and surface modules). Confocal images were denoised with despeckle filter and a background correction was applied by using FIJI software. The Imaris filament tracer pipeline can be applied upon background subtraction to reconstruct neuron morphology by using Imaris Bitplane software.

#### Imaging Using Inverted Microscopy Multiplexed at 96 or 384 Samples

5. Choose a lens with large working distances and adjustable RI. Match the RI of the lens to match RapiClear^TM^.
6. To ensure the minibrains are close to the imaging surface, remove 30 μl of RapiClear^TM^ from each well for a 96-well plate and 30 μl of RapiClear^TM^ from each well for a 384 well plate.
7. Proceed for imaging using required Z step size, 3 μm for volume rendering, and 10 μm for profiling the minibrain for signal.
  **Troubleshooting:** If the imaging is blurry, choose appropriate RapiClear^TM^ or change the objective with the correct RI to match the refractive indices of both.

## Timing

### Generation of Minibrains

I. Thawing frozen aliquots of NSC^hiPS^
  Step 1–10: 1 h and 30 min
II. Passaging and expansion of NSC^hiPS^
  Step 13–20: 1 h and 30 min
III. Week 0: Formation of spheres (3D)
  Step 22–26: 1 h
IV. Week 1: Differentiation phase I
  Step 27–28: 30 min
V. Week 2–4: Differentiation phase II
  Step 29: 15 min for one plate
VI. Week 3–5: Shifting to maintenance phase of the minibrains
  Step 30: 15 min for one plate.

### ALI Culture of Minibrain on Confetti

Step 1–5: 30–45 min for one plate.

### Viral Labeling of Projection Neurons in Live Minibrains

Step 1–8: 30 min

Step 9–10:2 h and 15 min

Step 11: 48 h.

### Immuno-Histochemical Staining of Minibrains

I. Minibrain fixation
  Step 1–2: 1 h 30 min
II. Antigen retrieval
  Step 3–4: 1 h 15 min
III. Permeabilization and blocking of the minibrain
  Step 5–6: 5–18 h
IV. Primary and secondary antibody labeling
  Step 7–10: 3 days
V. Nuclei staining with DAPI.
  Step 11–12: 4 h

### Minibrain Preparation for Microscopy

I. Permeabilization of minibrain for tissue clearing
  Step 1: 1 h 30 min
II. Mounting and clearing minibrains for 3D confocal imaging for upright confocal microscopy
  Step 2–6: 20 min to mount 3 minibrains per well onto 9 wells of the sample holder.
  Step 7: 24 h
III. Minibrain preparation for 3D imaging using inverted fluorescence microscopy
  Step 8–9: 3 min for one well.

### Imaging Minibrains

I. Imaging in upright confocal microscopy
  Step 1–3: 20 min for mounting 3 minibrains per well in 9 wells of the sample
  holder.
  Step 4: 30 min to 2 h for one minibrain
II. Imaging in inverted microscopy multiplexed at 96 or 384 samples
  Step 6: 1 min for 1 well
  Step 7: 10–30 min per minibrain (250 μm of imaging).

## Anticipated Results

Minibrain is a brain spheroid model, a complex ensemble of various types of excitatory neurons, inhibitory neurons and glial cells (Figure 4). The protocol allows generation of minibrains in large numbers and longitudinal maintenance of minibrain for long periods of time. The protocol generates minibrains ranging approximately between 500 and 600 μm, allowing viability of the minibrain for over 15 months. The viral labeling protocol is easy and quick, allowing visualization of neurons as early as 24 h after infection. Projection neurons with different morphologies are labeled through the protocol (Figure 7 and Supplementary Figure 3). Immunohistochemistry protocol is optimized to enable complete penetration of antibodies across the minibrain (Figure 8). The clearing of minibrains is suitable for lightsheet, upright and inverted microscopy allowing imaging of minibrains in 3D (Figures 6–8 and Supplementary Videos 1–5). The protocol allows complete imaging of the minibrain from which whole neuron morphology can be reconstructed (Figure 7 and Supplementary Figure 3). Using Imaris, neuron morphology properties can be extracted and analyzed (Figure 7). The protocol allows 3D rendering of minibrain immunohistochemistry allowing assessment of anatomical distribution of markers across the minibrain (Figure 8).

## Limitations

The viral labeling protocol we developed is currently limited to strong ubiquitous CAG promoters. Further testing is required to check efficiency of weak promoters that would allow labeling of specific cell types. The time required for imaging the minibrains in their whole thickness using a laser scanning technique can take up to 1–2 h when using a step size of 3 μm. This limitation can be circumvented by the use of lightsheet technology. We show that by using a clarity optimized lightsheet system (COLM), aggregates of minibrains can be quickly imaged all at once (Supplementary Video 2). Localizing single minibrains and vertically mounting minibrains is complicated and not convenient, hence it is difficult to multiplex imaging using this kind of light sheet microscopy set up. Further protocol establishment is required for light sheet imaging of minibrains. Dispensing minibrains to 96-well and 384 well plates for assays is time consuming and careful attention is required to avoid mix-up of the samples. This limitation can be partly overcome by using multi-channel pipettes and using printed sample/reagent layouts while dispensing minibrains and performing any further assays. For large screening studies, we show that clarified minibrains can be imaged using 384 well imaging culture plates (Supplementary Video 5) but images can be clearly obtained only until partial depth due to the quality of the lens used that is not optimized for tissue clarified samples.

## Discussion

Minibrains are an excellent choice of *in vitro* models to study early human neuronal development, aspects of gliogenesis, neurogenesis and neuronal connectivity. The iPSCs technology allows us to generate patient-specific minibrain models for various neurological disorders (Costamagna et al., 2019). Minibrains are cost-efficient, reliable and reproducible for testing drug therapeutic options, screening for toxicological effects and assaying for biocompatibility of human neural tissue (Sandström et al., 2017). The ability to maintain the minibrains for over a year allows the researcher to monitor and follow neuronal differentiation and network maturity longitudinally over time.

In this study, we present methodology for mass generation and maintenance of minibrains for large scale studies. In our methodology we adopt generation of minibrains from NSC^hiPSC^ instead of directly from iPSCs to circumvent multiple stem cell differentiation steps. We follow a slow progressive differentiation protocol that was modified from Sandström et al. (2017) published from our lab which allows us to generate relatively smaller brain spheroids in comparison to the brain organoid protocols (Paşca, 2016; Bagley et al., 2017; Lancaster et al., 2017; Xiang et al., 2017; Karzbrun and Reiner, 2019). The use of breathable, adhesive seals in our protocol reduces the frequency of medium changes and increased humidity maintenance, assuring better health of minibrains and longitudinal maintenance for over 15 months and above. ALI maintenance of minibrain allows integration of minibrains on micro-electrode array biochips and test biocompatibility of neural tissue on neuroprosthetic devices.

In this study, we present novel methodologies to study minibrains using neuronal labeling, immunohistochemistry, clearing and 3D imaging at multiplexed scales. The development of a custom multi-sample holder for the clarity confocal modules enables to image up to 9 wells, and at least 5 minibrains per well. Using immunohistochemistry and 3D imaging, we show that unlike cerebral organoids, in minibrains the progenitors (expressing Ki67) are not distributed to a central core but spread throughout the minibrains (Figure 8). Using our pipeline, we were able to confirm the presence of POU3F2 positive neurons and its distribution across minibrains in 3D (Supplementary Figure 4 and Figure 8). With our imaging pipeline, 2D imaging or partial 3D imaging of 3D brain models can be multiplexed for up to 96 or 384 samples using inverted microscopy, investing in long distance lenses, could possibly allow whole mount imaging using inverted microscopy (Supplementary Video 5).

It is not yet well understood how neurons shape their morphology in 3D *in vitro* brain models where spontaneous neuronal activity-based networks are the main source of input and where anatomical distribution of molecules like in human developing brains is absent. Thus, studying neuron morphology in 3D brain *in vitro* models, will allow us to study intrinsic self-organizing mechanisms that guide neuron morphology across distinct neuronal subtypes. By combining viral labeling, tissue clearing and confocal imaging, we were able to produce high resolution imaging dataset from whole minibrains revealing diverse neuron morphology reconstructions. By using Imaris Filament tracer we were able to segment 3D labeled cells and model neuronal dendrites allowing us to map various dendrite morphometric properties of neurons. We were able to confirm both long and short projecting neurons in minibrains using our pipeline. Combining neuronal markers with our protocol will allow users to reconstruct neuron morphology specific to distinct subtypes of neurons in minibrains.

More light sheet microscopes and 3D imaging systems have been recently developed for the purpose of imaging small biological samples at multiplexed scales (Alladin et al., 2019; Rakotoson et al., 2019). Our pipeline can easily be adapted for light sheet microscopy systems that have been designed for imaging small-sized biological samples and organoids, which will allow easy multiplexing and shorter time scales for imaging. We show by light sheet imaging of aggregates of minibrains that our pipeline can also be extended to larger brain organoids (Supplementary Video 2). In prevue of the fact that most researchers do not have access to light sheet microscopes, and the prevalence of the confocal microscopes our protocol will allow many researchers to image *3D brain in vitro* models.

In summary, the novel methodologies presented here will serve as a blueprint in using minibrains for large scale screening and modeling studies for the purpose of studying neuronal disorders, drug testing and chemical screening.

## Data Availability Statement

The original contributions presented in the study are included in the article/Supplementary Material, further inquiries can be directed to the corresponding author/s.

## Author Contributions

SG and AR designed, conceived the project, and developed the protocols and pipelines. SG, LS, and SO performed the experiments. SG, SO, and LB performed the imaging. SG and LB performed the image processing and analysis. SG wrote the manuscript. LS and AR supervised the project and provided critical inputs. All authors assisted in the preparation of the manuscript.

## Conflict of Interest

SG was employed by HEPIA and is curently employed by company ARMIA Lifesciences PVT Ltd. The remaining authors declare that the research was conducted in the absence of any commercial or financial relationships that could be construed as a potential conflict of interest.
