# Supplementary material for: Mass Generation, Neuron Labeling, and 3D Imaging of Minibrains
Source: Front Bioeng Biotechnol. 2021 Jan 7;8:582650. doi: 10.3389/fbioe.2020.582650 (PMC7883898; doi:10.3389/fbioe.2020.582650)
Supplement: Supplementary file 1 [file Data_Sheet_1.pdf]

Supplementary material and methods

Shallow RNA sequencing of the minibrains:

For the RNA extraction: Multiple minibrains from one well were transferred in a 1.5 ml microtube across four replicates and 500 µl of RNA protect cell reagent (Qiagen, #76526) was added.1 million NSC^{hiPSC} cells were collected across 4 replicates and 500 µl of RNA protect cell reagent (Qiagen, #76526) was added. Samples were disrupted and homogenized immediately using supplier protocol from the RNeasy® Plus Mini Kit (Qiagen, #74134,). After this RNA concentration was measured using Qubit 2.0 Fluorometer (Invitrogen, #Q32866) following supplier protocol. RNA sequencing library was prepared using Bulk RNA Barcoding and sequencing (BRB-seq) library preparation as described in Alpern et al., 2019 for 4 minibrains and NSC^{hIPSC} replicates. We next performed sequencing at low sequencing depth at an average of 1 million reads per sample. Previous studies have utilized shallow RNA sequencing to understand various biological models (Atallah et al., 2013; Heimberg et al., 2016). Differential gene expression analysis and gene ontology analysis was performed using iDEP 9.2 platform and was able to identify biological processes differentially regulated between minibrains and NSC^{hIPSC} (Ge et al., 2018).

Electrophysiology:

Electrophysiological data from minibrain was obtained using a customized Micro-Electrode Array (MEA) biochip platform made by HEPIA including a porous membrane MEA specifically designed for air-liquid interface type of culture with a total of 4x8 electrodes, 8 electrodes being dedicated per minibrain. We used platinum electrode coated with black platinium with a diameter of 30 µm and an inter-electrode distance of 200 µm. A mini-fluidic system was implemented to the MEA-biochip that allows an automatic perfusion of the culture medium. A customized data acquisition software was developed to record and extract action potentials from the raw data by a thresholding method (Figure 3D). Detected action potentials (Figure 3E) were then represented as a “time-stamp” graph (Figure 3F).

Light sheet microscopy:

Light-sheet imaging was performed using a customized version of the Clarity Optimized Light-sheet Microscope (COLM)2 at the Wyss Center Advanced Light-sheet Imaging Center, Geneva. Briefly, the sample was illuminated by one of the two digitally scanned light sheets. Emitted fluorescence was collected by a 10X XLFLUOR4X N.A. 0.6, filtered (609/54 nm, Semrock BrightLine HC) and imaged on an Orca-Flash 4.0 LT digital CMOS camera at 4 frames per seconds.

Golgi-Cox staining of whole mount organoids:

The Ultra-rapid Golgi stain (URG) solution was prepared as described by Kassem, 2018. Minibrains were stained with URG solution in a glass vial in dark at 42°C for 24 hours. Minibrains were then washed twice in distilled water and incubated in 30% ammonium hydroxide for 20 minutes. Minibrains were then rinsed once in distilled water and were incubated in 10% sodium thiosulfate for 20 minutes. Minibrains were then rinsed briefly in distilled water and incubated in DAPI (10 µg/ml in PBS with 0.1% Triton X100) for an hour. Minibrains were then washed with PBS and mounted for clearing and 3D imaging. Imaging was performed with Zeiss LSM 880 confocal microscope with reflection imaging using the 488 nm wavelength, as described in Kassem et al., 2018.

Image processing:

To perform image analysis and 3D rendering on the Z-stacks, FIJI or Imaris software (filament tracer and surface modules) were used. The automatic filament tracer pipeline was applied upon background subtraction to reconstruct neuron morphology in Imaris software. Dendritic properties for Sholl analysis, number of terminal points, total dendritic lengths and branch points were retrieved from the neuronal 3D reconstructions using the Imaris software.

Counting cells:

The position of stained cells ($Ki67^+$ or $POU3F2^+$) and DAPI cells was grouped based on their X, Y, Z coordinates in 10 concentric circles across the different radius of the sphere. The ratio of stained cells to DAPI cells in each circle was calculated as a percentage.

Construction of 3D imaging microscopy support:

ABS-P430™ XL Model (Ivory) (Stratasys, #345-42005)
3D printer SR-30™ XL Soluble support (Stratasys, #345-42207)
The microscopy support for the sample holder was 3D printed with Uprint Stratasys 3D printer using ABS material and soluble support.
Please contact corresponding author for 3D printing source file.

Preparation of sample holder using laser cutting:

Materials:

- 2 mm Poly(methyl methacrylate) (PMMA) plate (TopAcryl Hesaglas, #VOS120000000)
- Double-sided adhesive (3M, #467MP)
- Laser cutter (Trotec, Speedy 100R)

Steps:

1. Remove the protective film on one side of the PMMA plate and apply the double-sided adhesive.
2. Repeat step 1 on the other side of the PMMA plate.
3. Place the prepared PMMA plate in the laser cutter.
4. Turn on the laser cutter.
5. Focus the laser beam on the PMMA plate and launch the software.
6. Connect the software to the laser cutter.
7. Place the laser on the desired cutting space and create an anchor point of the position in the software's workspace.
8. Import the file "adapt_Minibrain_conf_2mm_cut_and_467mp_precut.svg".
9. Drag the imported cutting procedure in the software's workspace and set the power to "PMMA VOS 2mm" from the HEPIA's directory.
10. Vectorize the cutting job.
11. Anchor the cutting job to the previously made anchor point.
12. Estimate the cutting times and launch the cutting job.
13. Allow around 1 minute to extract the gases after the end of the cutting job prior opening the hood.

14. Flip the cut piece without moving the PMMA plate.
15. Repeat steps 8 to 13 using the file “adapt_Minibrain_conf_467mp_precut_only.svg”
16. Remove the cut piece and the PMMA plate from the laser cutter.
17. Repeat steps 8 to 16 to obtain the desired number of pieces.
18. Degas the pieces using an oven at 80°C for around 72 hours.
19. The pieces are ready to be used

Note:

- Contact adrien.roux@hesge.ch for “adapt_minibrain_conf_2mm_cut_and_467mp_precut.svg” and “adapt_minibrain_conf_467mp_precut_only.svg” files.
- Store the pieces in an enclosed space to prevent dust buildup
- 3D rendering of the sample holder can be visualized using PTC creo software.
  adapt_minibrain_conf.prt.5
  Asm_adapt_minibrain_conf.asm.1
  coverslip_18mm.prt.2
  Contact adrien.roux@hesge.ch for the files to print the sample holder and to visualize it.

References:

Alpern, D., Gardeux, V., Russeil, J., Mangeat, B., Meireles-Filho, A. C. A., Breysse, R., et al. (2019). BRB-seq: ultra-affordable high-throughput transcriptomics enabled by bulk RNA barcoding and sequencing. *Genome Biol* 20, 71. doi:10.1186/s13059-019-1671-x.

Atallah, J., Plachetzki, D. C., Jasper, W. C., and Johnson, B. R. (2013). The Utility of Shallow RNA-Seq for Documenting Differential Gene Expression in Genes with High and Low Levels of Expression. *PLOS ONE* 8, e84160. doi:10.1371/journal.pone.0084160.

Ge, S. X., Son, E. W., and Yao, R. (2018). iDEP: an integrated web application for differential expression and pathway analysis of RNA-Seq data. *BMC Bioinformatics* 19, 534. doi:10.1186/s12859-018-2486-6.

Heimberg, G., Bhatnagar, R., El-Samad, H., and Thomson, M. (2016). Low Dimensionality in Gene Expression Data Enables the Accurate Extraction of Transcriptional Programs from Shallow Sequencing. *cels* 2, 239–250. doi:10.1016/j.cels.2016.04.001.

Supplementary Figures

Supplementary Figure 1: Troubleshooting and monitoring of minibrain maturation and viability.

(A) Shows sealing of a cell culture plate with a breathable sealer to avoid media evaporation. **(B)** Left and center images shows fusion of organoids that indicate incomplete differentiation of minibrains, in which case the minibrains should not be

transferred to NDM medium immediately. Image on the right shows unfused minibrains obtained upon complete differentiation. **(C)** Shows how to assess minibrain viability.

Supplementary Figure 2: Mounting organoids for 3D imaging.

(A) Shows the geometry of the sample holder. **(B)** Shows mounting of minibrains onto the sample holder. **(C)** Shows mounting of sample holder with minibrains on microscope support for confocal imaging using the LSM 880 Zeiss microscope.

Supplementary Figure 3: Diversity in neuron morphology in minibrains.

(A) Left shows maximum intensity projections of 3D image of minibrains containing tdTomato expressing neurons (labeled through AAV^{RG} viral particle infection containing tdTomato transgene, Age of minibrain = 10 weeks), Right shows select representative neuron morphology traces from the minibrains reconstructed with Imaris segmentation pipeline. **(B)** Left shows whisker plots of Sholl analysis performed on a total of 23 neurons obtained from 3 minibrains pooled together, right shows the same data displayed as jitter plot (each dot corresponds data from one neuron).

Videos

Supplementary Video 1:

Volume rendered and segmented 3D image of Golgi-Cox stained minibrains

Supplementary Video 2:

Light sheet microscopy 3D imaging of aggregated minibrains

Supplementary Video 3:

3D image of viral labelled neurons in 3-month-old minibrains

Supplementary Video 4:

3D image of Ki67 staining of 1-week-old minibrain

Supplementary Video 5:

3D image of DAPI labelled 5-month-old minibrain in inverted microscope with 20X air objective, scale in µm.

Tables

Supplementary Table 1

Cost of minibrain generation

Supplementary Table 2

Dendrite morphometric analysis for figure 7E

Supplementary Table 3

Sholl analysis data for Figure 7D

Supplementary Table 4

Dendrite terminal point distribution across neurons for Figure 7F

Supplementary Table 5

Distribution of progenitors across 1 minibrain for Figure 8D

Supplementary Table 6

Distribution of POU3F2 positive neurons across 1 minibrain for Figure 8E

Supplementary Table 7

Table contains fold change and adjusted p value for the list of genes that were differentially regulated between minibrain and NSC^{hIPSC}. The expression values were used to generated the heatmap in Figure 4A.
