# Supplementary figures and images for: Mass Generation, Neuron Labeling, and 3D Imaging of Minibrains
Source: Front Bioeng Biotechnol. 2021 Jan 7;8:582650. doi: 10.3389/fbioe.2020.582650 (PMC7883898; doi:10.3389/fbioe.2020.582650)

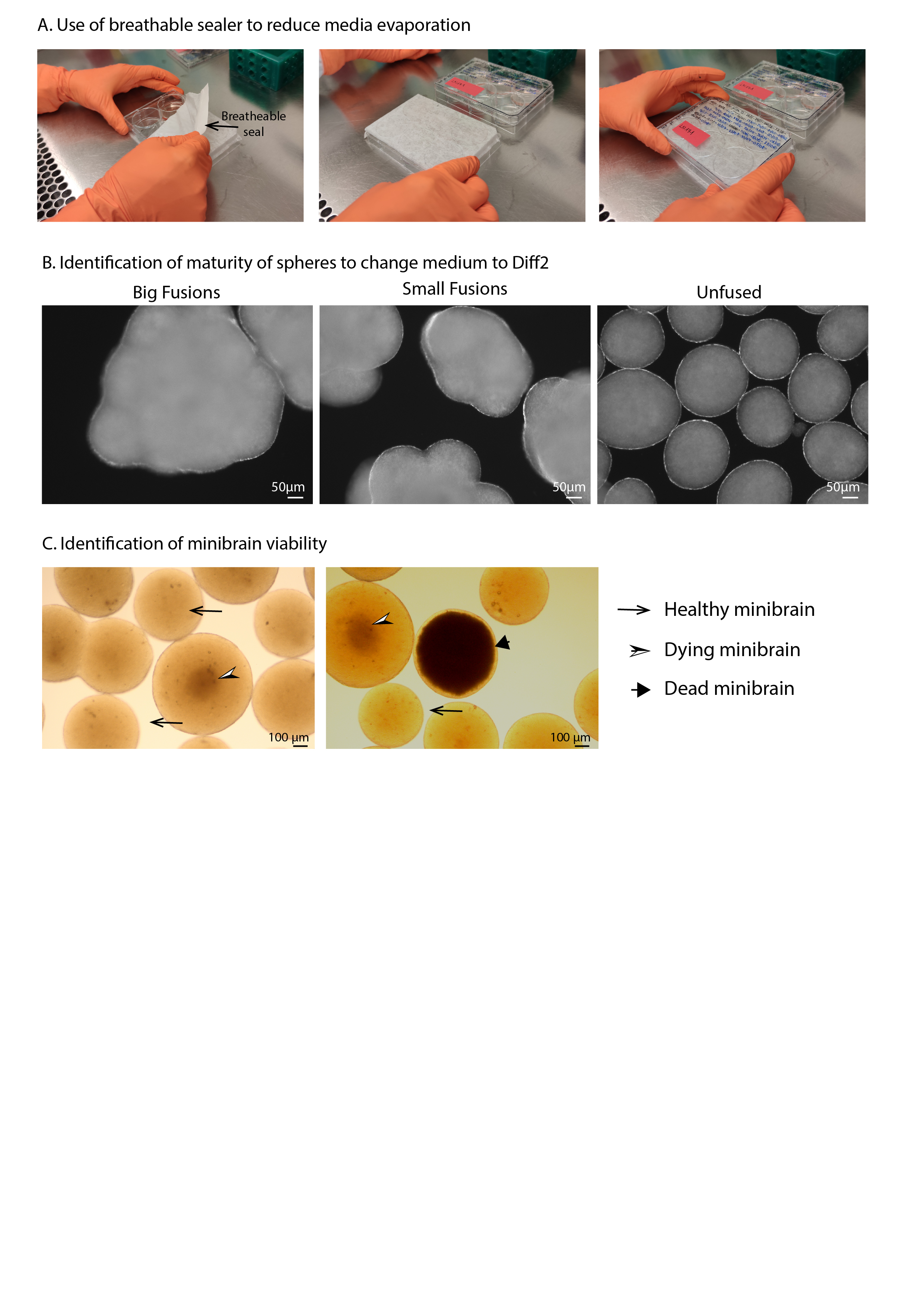

Supplement: Supplementary file 2 [file Image_1.jpg]

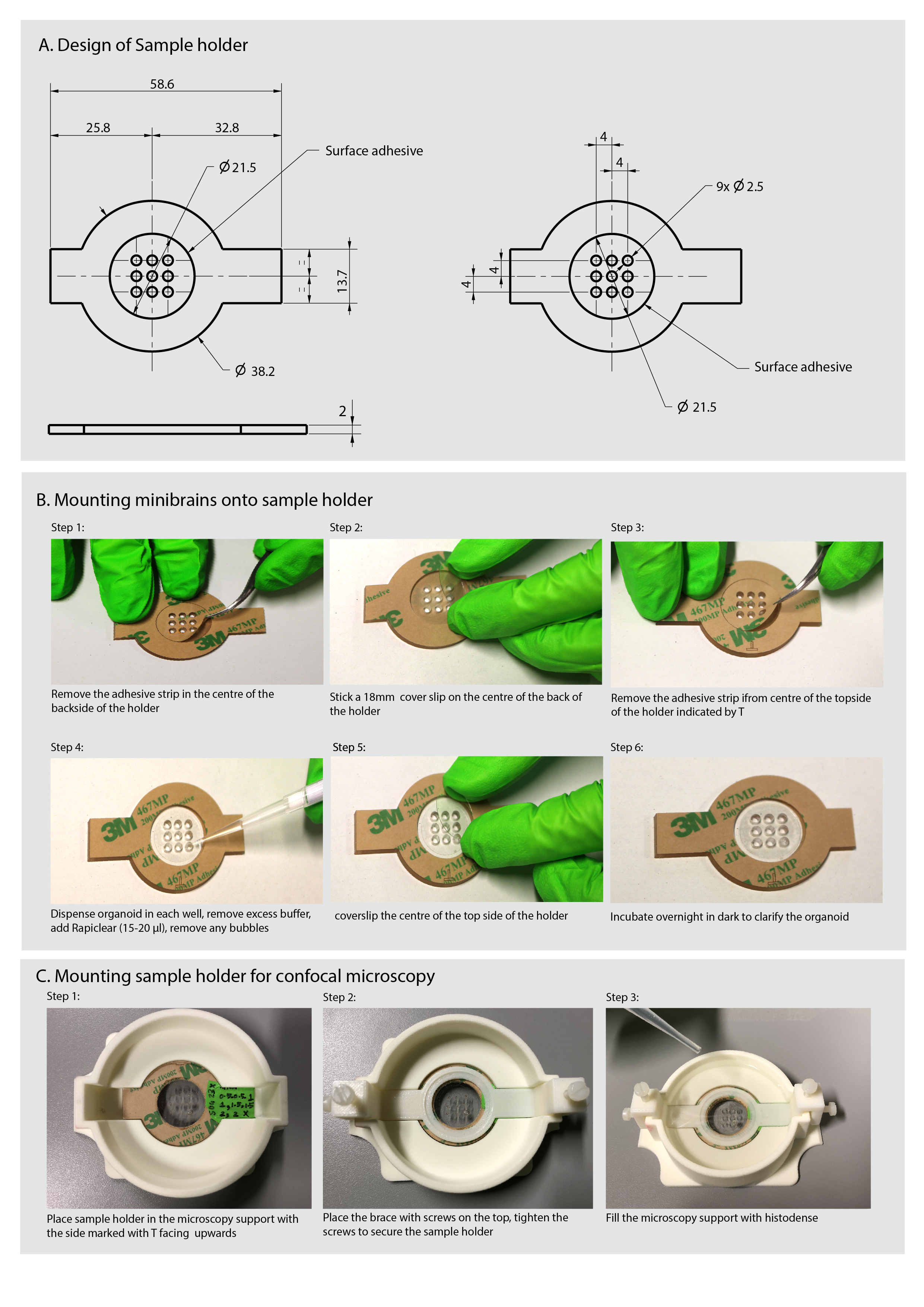

Supplement: Supplementary file 3 [file Image_2.jpg]

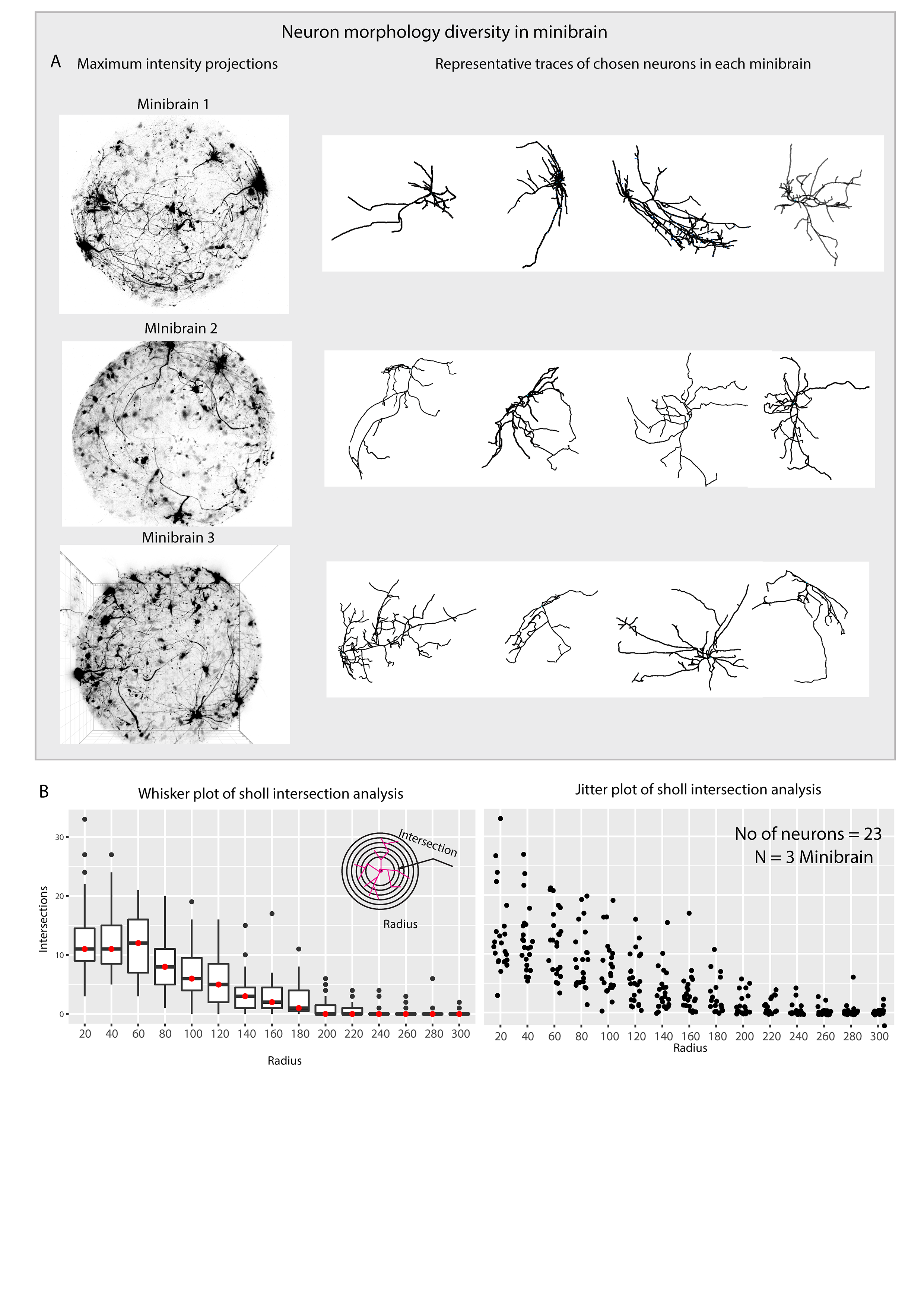

Supplement: Supplementary file 4 [file Image_3.TIF]
